# Co-occurrence of diabetes, myocardial infarction, stroke, and cancer: quantifying age patterns in the Dutch population using health survey data

**DOI:** 10.1186/1478-7954-9-51

**Published:** 2011-09-01

**Authors:** Pieter H van Baal, Peter M Engelfriet, Hendriek C Boshuizen, Jan van de Kassteele, Francois G Schellevis, Rudolf T Hoogenveen

**Affiliations:** 1 Expertise Centre for Methodology and Information Services, National Institute for Public Health and the Environment (RIVM), Bilthoven, the Netherlands; 2 Institute of Health Policy & Management/Institute for Medical Technology Assessment, Erasmus University Rotterdam, Rotterdam, the Netherlands; 3 Centre for Prevention and Health Services Research, National Institute for Public Health and the Environment (RIVM), Bilthoven, the Netherlands; 4 Netherlands Institute for Health Services Research (NIVEL), Utrecht, the Netherlands; 5 Department of General Practice/EMGO+ Institute, VU University Medical Center Amsterdam, Amsterdam, the Netherlands

**Keywords:** multimorbidity, comorbidity, diabetes, cancer, cardiovascular disease, stroke, P-splines

## Abstract

**Background:**

The high prevalence of chronic diseases in Western countries implies that the presence of multiple chronic diseases within one person is common. Especially at older ages, when the likelihood of having a chronic disease increases, the co-occurrence of distinct diseases will be encountered more frequently. The aim of this study was to estimate the age-specific prevalence of multimorbidity in the general population. In particular, we investigate to what extent specific pairs of diseases cluster within people and how this deviates from what is to be expected under the assumption of the independent occurrence of diseases (i.e., sheer coincidence).

**Methods:**

We used data from a Dutch health survey to estimate the prevalence of pairs of chronic diseases specified by age. Diseases we focused on were diabetes, myocardial infarction, stroke, and cancer. Multinomial P-splines were fitted to the data to model the relation between age and disease status (single versus two diseases). To assess to what extent co-occurrence cannot be explained by independent occurrence, we estimated observed/expected co-occurrence ratios using predictions of the fitted regression models.

**Results:**

Prevalence increased with age for all disease pairs. For all disease pairs, prevalence at most ages was much higher than is to be expected on the basis of coincidence. Observed/expected ratios of disease combinations decreased with age.

**Conclusion:**

Common chronic diseases co-occur in one individual more frequently than is due to chance. In monitoring the occurrence of diseases among the population at large, such multimorbidity is insufficiently taken into account.

## Introduction

The prevalence of chronic diseases has increased strongly the last few decades in most Western countries [1,2]. Besides aging of the population, this is also partly due to increased survival in people with many chronic conditions [3,4]. Given the high prevalence of chronic diseases, it is not surprising that the presence of multiple chronic diseases within one person has also become more common [5]. This phenomenon is known as multimorbidity, or as comorbidity if one disease is considered as the primary, or index, condition [6]. Even if we assume that diseases are distributed randomly and occur independently of each other, we expect a great share of multimorbidity at older ages [7,8]. For instance, if 20% of those 65 years or older suffer from diabetes mellitus (DM) and if the prevalence of osteoarthritis is 20% in this group, 4% will suffer from both diabetes and osteoarthritis by sheer coincidence. Clustering of diseases in individuals is to be expected for several reasons [6,9]. First, as mentioned, on the basis of coincidence, and second, because some diseases are known to be causally related. For instance, diabetes is a risk factor for acute myocardial infarction (AMI) and stroke (cerebrovascular accident [CVA]) and, therefore, these diseases will be more common among diabetics. Thirdly, clustering of diseases can result from the presence of common underlying known or unknown risk factors, as many risk factors (e.g., smoking and BMI) are related to multiple chronic diseases. And finally, diseases tend to cluster within individuals due to differences in individual susceptibility to disease. In elderly people, this is often referred to as frailty [10].

Multimorbidity is often used as an explanatory variable in research to adjust for "case mix," or as a determinant of prognosis of the main disease of interest [11]. However, the view that multimorbidity is an object of study in itself is gaining support [9,12]. Since people with multimorbidity have an increased mortality risk, higher health care utilization, and greater quality of life losses than people with a single disease [13], any description of the distribution of diseases in the population at large is incomplete without estimates of how often combinations of chronic diseases occur. Moreover, as the co-occurrence of common chronic diseases is more frequent than is to be expected on the basis of chance, monitoring of the prevalence of multimorbidity seems a logical thing to do in an aging society. In this manner, it might be possible to better identify groups at increased risk, to identify new risk factors that specifically apply to comorbidity, and thus to devise appropriate public health interventions. Furthermore, given the current attention on medical guidelines and disease management programs, it is crucial to take multimorbidity into account [9,14,15].

The associations between some specific pairs of diseases have been investigated in more detail, in particular causally-related diseases. Thus, in numerous studies the occurrence of diseases, such as coronary heart disease and stroke in diabetics, has been investigated [16-18]. Much less is known about the clustering of other disease combinations, such as heart disease and cancer, in the general population. Another issue that has rarely been addressed explicitly is the role of age [19]. Even though it is to be expected that the prevalence of multimorbidity increases with age, it would be interesting to gain more insight into the nature of this relation, and how this, in turn, relates to the age-dependence of the prevalence of the individual diseases. In this article we compare the joint occurrence of pairs of four of the most prevalent chronic diseases (diabetes, AMI, cancer, and stroke), and we focus especially on the role of age. We estimate to what extent specific pairs of diseases cluster within people and how this deviates from what is to be expected under the assumption of independence.

## Methodology

### Data

We used data from the Permanent Survey of Living Conditions (POLS: Permanent Onderzoek LeefSituatie) covering the years 2001 to 2007. POLS is an ongoing yearly cross-sectional survey, started in 1981 and coordinated by Statistics Netherlands [20]. The POLS survey data, which require no ethics approval, is publicly available from http://www.dans.knaw.nl. POLS monitors developments in lifestyle, health, medical consumption, preventive behavior, and well-being in the Netherlands. Before 1997, the surveys used to be sampled with households as the underlying unit. Since 1997, surveys have been sampled on the basis of person records from a centralized municipal registry. The interviewer visits the participants at home, asks for informed consent and leaves a written (drop-off) questionnaire. Yearly net participation currently ranges around 10,000 individuals, with response percentages of around 60%. In the POLS surveys in the years 2001 to 2007, the following questions on disease status were included:

1. Diabetes: Do you have diabetes?

2. Stroke: Did you ever experience a stroke, cerebral hemorrhage, or cerebral infarction?

3. AMI: Did you ever experience a myocardial infarction?

4. Cancer: Did you ever have a cancer?

With four diseases included, there are a total of six different pairs of diseases: diabetes and AMI, diabetes and stroke, diabetes and cancer, AMI and stroke, AMI and cancer, and stroke and cancer. Table 1 displays characteristics of the survey for the different years.

**Table 1:** Characteristics of the POLS survey, 2001-2007

*Year*	*N *	*% Men*	*% Diabetes *	*% Stroke *	*% AMI *	*% Cancer *
2001	9676	49.2	3.4	1.7	2.6	4.4

2002	9744	48.3	3.2	2.1	3	3.9

2003	9877	49.3	3.6	2.3	2.9	4.6

2004	11117	48.9	3.9	2	3	5.4

2005	10378	48.9	4.4	2.4	3.1	6

2006	9607	49.0	4.4	2.4	2.8	5.5

2007	8741	48.3	4.8	2.5	2.9	5.9

2001-2007	69140	48.9	4.0	2.2	2.9	5.1

## Methods

**Figure 1:**
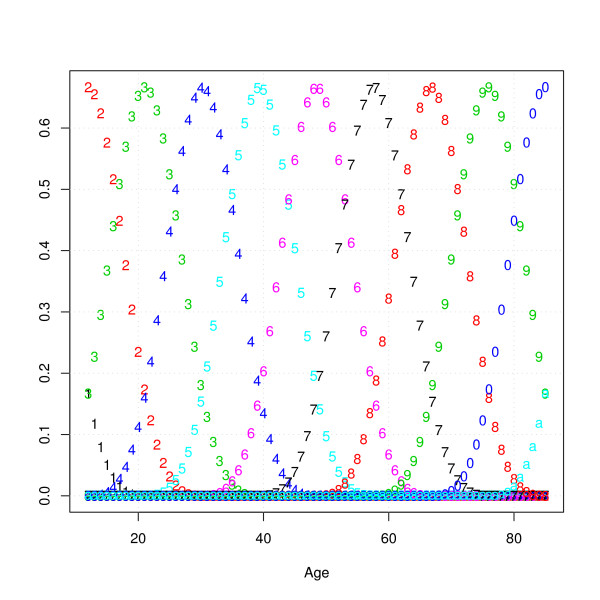
**Cubic B-spline basis functions used for P-spline smoothing (each number indicates a different B-spline basis function)**.

For each combination of two diseases (disease A and disease B), a variable was created that could take on the following values: 0 (no disease), 1 (only disease A), 2 (only disease B), 3 (both diseases). These variables were entered as the dependent variable in a multinomial regression simultaneously estimating the following probabilities: P(no A, no B), P(A, no B), P(no A, B), and P(A, B). In order to derive a smooth relation between age and these probabilities from the rough data, we used P-spline smoothing [21]. P-splines are a combination of B-splines and penalized regression. The method may be described briefly as follows. First, one defines a large number of equally-spaced cubic B-spline functions over the age interval. B-splines are polynomial functions that have a non-zero value only within a specified range. Figure 1 displays the cubic B-spline basis functions used in our analyses, which are equally spaced nonoverlapping third order polynomial functions.

A key feature of cubic B-splines is that any linear combination of the basis functions will result in a smooth function with a second-order derivative that is continuous at the joining points. Cubic B-splines share the advantage of dummy variables (local basis) and polynomials (smoothness) without their disadvantages. For dummy variables, a disadvantage is that the age gradient would not be smooth, while with polynomials, values at high ages can strongly influence the fit at lower ages. The drawback of B-splines and other forms of local regression is that it is difficult to determine the number of knots and spacing of the basis functions. As a solution to this problem, P-splines were proposed. The general idea behind P-splines is to use a relatively large number of knots and to put a penalty on the difference between the coefficients of adjacent cubic B-spline functions. The optimal amount of smoothing in P-splines is then determined by adjusting the weight of the penalty using cross-validation or an information criterion. In our analyses, the optimal smoothing parameters were found by minimizing the Aikaike Information Criterion (in Additional file 1 results are presented when the Bayesian information is used instead). All analyses were done in R http://www.r-project.org

### Outcome measures

In this paper, we will focus on three outcome measures for which we will present age-specific estimates based on predictions of the six estimated multinomial regression models. First, we will present estimates of the prevalence of pairs of diseases. Second, to asses on an absolute scale to what extent co-occurrence cannot be explained by independent occurrence, we calculated observed minus expected co-occurrence for each pair of diseases in the following manner:

$$\begin{array}{l} Obs-Exp=\text{P(A,B)-P(A)*P(B)}\\ \;=\text{P(A,B)-\{P(A, no B)}+\text{P(A,B)\}*\{P(no A,B)}+\text{P(A,B)\}} \end{array}$$

with P(A, B) as the observed proportion and the expected proportion being P(A) times P(B). Expected prevalence of pairs of diseases was calculated on the assumption of independence. Third, to asses the relative deviation from independent co-occurrence, we estimated observed/expected co-occurrence ratios:

$$\begin{array}{l} Obs/Exp=\frac{\text{P(A,B)}}{\text{P(A)*P(B)}}\\ =\frac{\text{P(A,B)}}{\text{\{P(A, no B)}+\text{P(A,B)\}*\{P(no A,B)}+\text{P(A,B)\}}} \end{array}$$

Confidence intervals around these outcome measures were calculated using Monte Carlo simulations. Regression coefficients of the regression models were repeatedly drawn from a multivariate normal distribution (sample size was set at 10,000). For each draw of the regression coefficients, predictions were made and observed/expected differences and ratios were calculated. After all draws were performed, confidence intervals were obtained by taking the 2.5th and 97.5th percentiles of the outcome measures.

## Results

**Figure 2:**
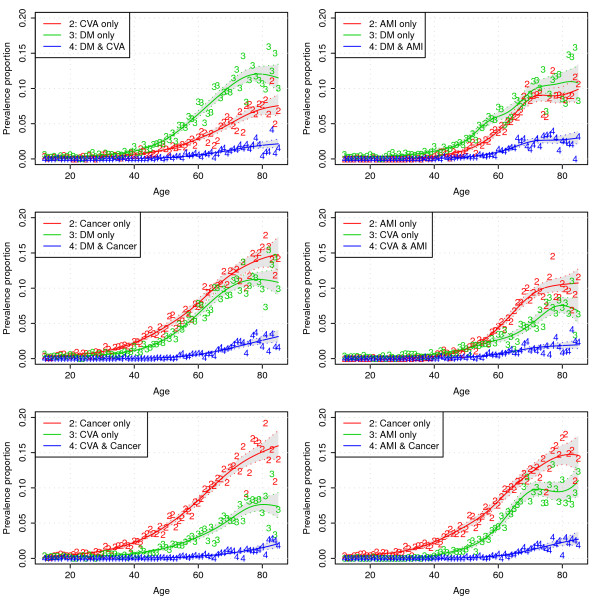
**Model predictions and data as a function of age for the six multinomial regression models (numbers indicate age-specific proportions observed in a particular year in the POLS survey)**.

Figure 2 displays the data and predictions of the six multinomial regression models that were estimated (it should be noted that we omitted the estimates for the level "none of the two diseases" from the figures, which is simply the complement of the other categories).

From Figure 2 it can be seen that, in general, prevalence for all diseases and disease combinations increases with age, but that the rate of increase is lower (or even negative) at higher ages. If we look, for instance, at the upper left panel of Figure 2, at age 80 years about 12% of people have diabetes without ever having experienced a stroke, about 7% of people have experienced a stroke but do not have diabetes, and about 2% of the 80 year olds have diabetes and a history of stroke.

**Figure 3:**
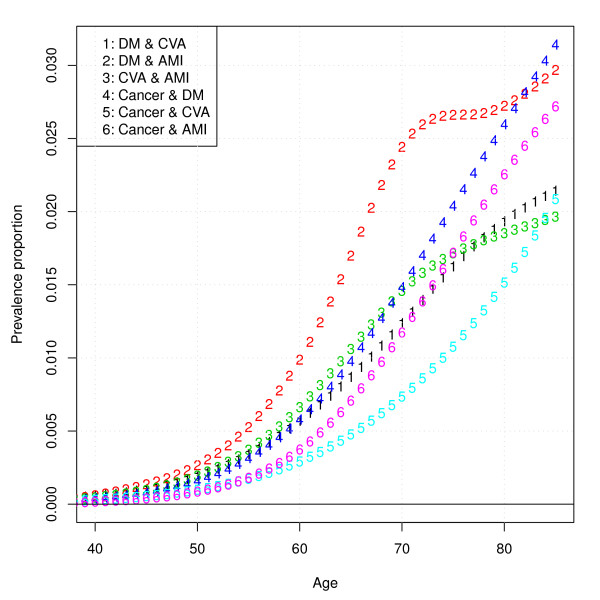
**Model predictions of prevalence proportion of all pairs of diseases specified by age**.

In Figure 3, the prevalence of all six pairs of diseases is displayed. From Figure 3, it can be seen that the prevalence of pairs of diseases increases with age and that the pair of greatest prevalence for most ages is diabetes in combination with AMI, and the pair of smallest prevalence is CVA in combination with cancer.

**Figure 4:**
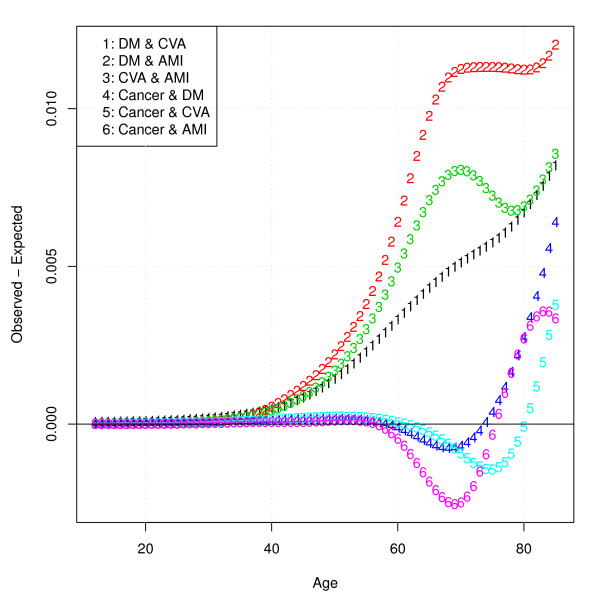
**Model predictions of observed disease pair prevalence minus expected disease pair prevalence**.

Figure 4 displays observed minus expected joint disease prevalence, indicating to what extent diseases co-occur more often than is to be expected under the assumption of independence. From Figure 4 it can be seen that for disease combinations without cancer, observed minus expected co-occurrence of disease pairs increases with age. For combinations of diseases including cancer, observed minus expected co-occurrence is negative in an age range roughly from 60 to 75 or 80 years. The absolute degree of "unexpected" co-occurrence is highest for the combination diabetes and AMI.

**Figure 5:**
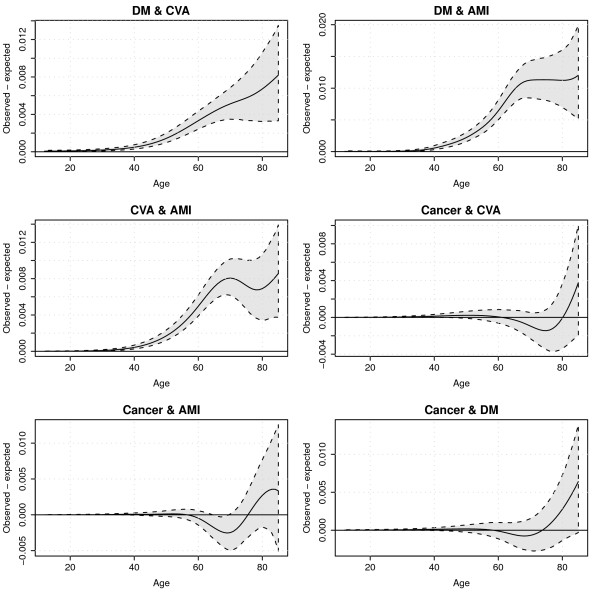
**Model predictions of observed disease pair prevalence minus expected disease pair prevalence with 95% prediction intervals**.

To appreciate the uncertainty surrounding this outcome measure, Figure 5 displays confidence intervals around observed minus expected co-occurrence for all disease pairs. What can be seen from Figure 5 is that uncertainty increases with the level of unexpected co-occurrence as it increases with age. The level of co-occurrence in the disease pairs including cancer often is not significantly different from zero.

**Figure 6:**
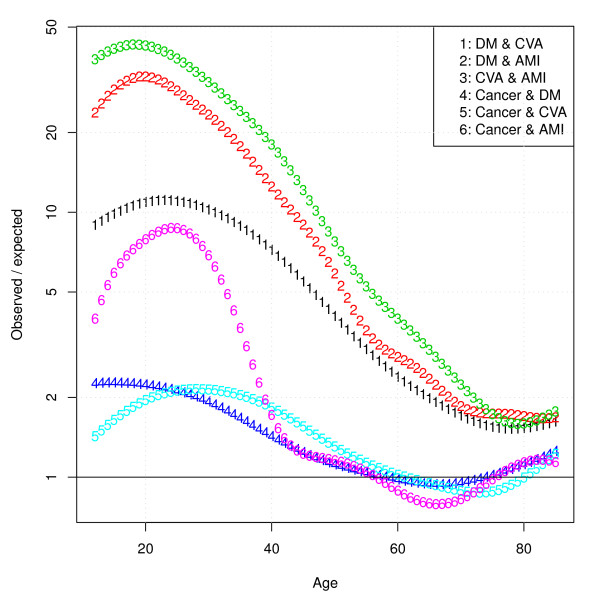
**Model predictions of observed/expected co-occurrence ratios**.

Figure 6 displays the ratios of the observed/expected joint prevalences. From this graph it can be seen that, although at lower ages co-occurrence of chronic diseases seems rare in an absolute sense, they tend to cluster much more, as can be inferred from the decreasing ratios. In line with Figures 4 and 5, the observed/expected ratios for disease pairs with cancer are less than one for the age range 60 to 80 years.

**Figure 7:**
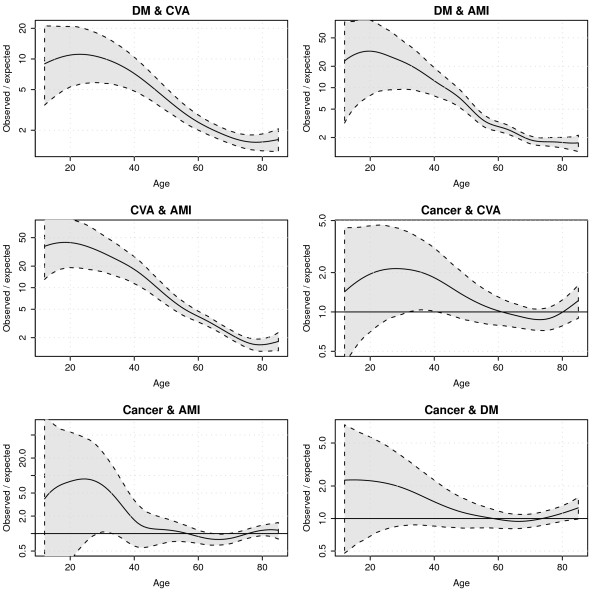
**Model predictions of observed/expected co-occurrence ratios with 95% prediction intervals**.

Figure 7 displays confidence intervals around observed/expected ratios for all disease pairs. What can be seen from this graph is that at low ages, uncertainty surrounding the ratios is very large due to a small number of cases, and uncertainty decreases at higher ages. In accordance with Figure 5, for most age ranges, ratios involving cancer were not significantly different from 1.

## Discussion and conclusions

In this study, we estimated the age-specific joint prevalence of all pairs of four of the most prevalent chronic diseases in the Dutch population. Co-occurrence of all disease pairs studied was seen to increase with age. The joint prevalence was highest for diabetes and AMI, while cancer and stroke co-occurred the least frequently. For all pairs not including cancer, co-occurrence was more frequent for all age groups than expected when the individual diseases occur independently. Thus, observed minus expected proportions increased with age, while the corresponding observed/expected ratios became smaller. This implies that although at lower ages co-occurrence is less prevalent, at lower ages chronic diseases tend to cluster more within individuals. Diabetes co-occurred frequently with stroke and AMI, which is in line with what is known about the increased risk for these diseases in diabetics. On a relative scale, as measured by the observed/expected ratio, stroke and AMI co-occurred most frequently. This is not surprising, as both events are related in their etiology and share multiple underlying risk factors such as high blood pressure, cholesterol, smoking, and obesity. Cancer, however, seemed to display a somewhat different behavior: within an age range of approximately 60 to 75 years, it co-occurred less frequently than expected with the other three diseases. This pattern was somewhat unexpected and not easy to interpret. On the face of it, this seems to imply that diabetes and cardiovascular diseases "protect" against cancer and/or vice versa. However, to the best of our knowledge, there is no known patho-physiological mechanism that could explain such a relation. Alternatively, it could be a "survivor" effect: those prone to develop both diseases die of cardiovascular disease before reaching the age at which cancer would become symptomatic. Yet another explanation might be that people adapt their lifestyles after being diagnosed with cancer. It needs to be stressed that the uncertainty in these estimates is large, and that the findings regarding cancer can be due to chance. Although interesting, at this point we cannot attach much significance to this observation.

A limitation of our study was that the institutionalized population was not included in the survey. As the prevalence of chronic diseases is probably higher among those institutionalized [22], this exclusion is likely to have led to some degree of underestimation of the prevalence of co-occurrence of chronic diseases. Furthermore, the response rate in this survey was not much more than 60%, which is a potential source of bias. Also, the self-reported nature of the data may have induced some bias in different ways. First, people might not accurately report their disease status. However, previous studies showed that self-reports of chronic conditions were fairly accurate, suggesting that this form of bias probably remained limited [23,24]. Second, if nonresponse was related to disease status, bias would result. To investigate whether this was the case, we compared our estimates to other national representative estimates of diagnosed disease prevalence [1,25,26]. Although estimates of cancer and diabetes prevalence were very similar, our estimates for AMI and stroke appear to be high. This could possibly be explained by a less-stringent case definition. Third, even if people report accurately and there is no selective nonresponse, undiagnosed cases will be missed. In case of diabetes, it has been argued that for every diagnosed diabetes case, there may be around 0.5 to one undiagnosed case. Thus, the true prevalences include, depending on the type of disease and other factors, variable proportions of people with no current morbidity or disability. Although there has been an upward trend in the ratio of undiagnosed/diagnosed cases of diabetes in the Netherlands [27], there are no recent observational studies in the Netherlands that have presented estimates of disease co-occurrence among diabetics. If there still is substantial underdiagnosis, we hypothesize that having a diabetes diagnosis is more likely in people with comorbidity. This would imply that our estimates of observed/expected ratios could be too high. Other limitations of our analyses are that cancer was treated as a single entity, whereas it is heterogeneous condition, and that no distinction was made between diabetes Types 1 and 2.

A few remarks are necessary regarding the method we used in modeling the joint presence of two diseases in the same individuals. Most importantly, we aimed at expressing prevalence as a function of age. With two diseases there are four possibilities. Hence, the outcome variable has a multinomial distribution, which we related to age using P-splines. The advantage of P-splines compared to polynomial regression is that model fit at the lower ages is not influenced by that at higher ages, and vice versa. That is, P-splines can be seen as a form of "local" regression. Furthermore, with P-splines it is not necessary to choose a more or less arbitrary number of knots, which is often seen as a drawback of other types of splines, such as B-splines. The choice of the smoothing parameter(s) for P-splines is data driven. In our analyses, we used the Akaike information criterion (AIC) criterion, which was also used by Eilers and Marx [21]. In Additional file 1, results are shown when the Bayesian information criterion (BIC) is used to find the optimal smoothing parameter. In general, the results are similar, but a bit smoother and less wiggly when the BIC criterion is used compared to the AIC criterion. For the absolute co-occurrence prevalence, the estimates do not differ much between the AIC and BIC. However, for the observed/expected ratios, there is a clear influence at lower ages, in which the prevalences are generally low. The observed/expected ratios at those ages are much higher if the BIC is used. Finally, it should be noted that in order to increase power, we combined both sexes and pooled all years. This means that the estimates are time- and gender-averaged. Stratifying the analyses by sex and analyzing time trends would therefore be a next step.

Although the "clustering" of chronic diseases is not a surprise, quantitative data on multimorbidity are scarce. Especially at older ages, the co-occurrence of chronic conditions starts to become so common that individuals with more than one disease can no longer be considered the exception. This not only has consequences for disease management programs, but also guidelines should more explicitly address the issue of comorbidity than has hitherto been done. The fact that the care for this category of patients poses specific difficulties requiring a distinctive approach is still insufficiently recognized. A better appreciation of the epidemiology of multimorbidity is a first step to bring the magnitude of the problem into focus. A noteworthy point of our study is that we have presented estimates combining cancer with noncancerous diseases. Although cancer incidence and prevalence are usually well-monitored by cancer registrations, these are not often linked to noncancerous diseases.

In conclusion, in this study we quantified age-specific co-occurrence patterns. It is clear that with increasing age, multimorbidity becomes common. More importantly, the prevalence of multimorbidity most of the time is much greater than would be the case if diseases occur independently from each other. Thus, the practice in epidemiological and public health research to monitor individual diseases tells only part of the story. With an aging population, it is important to quantify the problem of multimorbidity. Those involved in the management of care, the drafters of guidelines, and the doctors treating patients with more than one disease should develop strategies to improve the care for this category of patients that is becoming more numerous as the population ages.

## Competing interests

The authors declare that they have no competing interests.

## Authors' contributions

PHVB did the analyses and drafted the initial manuscript. JVDK, RTH, and HCB contributed to the development of the methodology. All authors contributed to the writing of the manuscript, read and approved the final manuscript.

## Supplementary Material

Additional file 1 **Additional file **1**shows the results if the Bayesian information criterion (BIC) is used to find the optimal smoothing parameters for the P-splines**. Click here for file
