# Supplementary material for: Co-occurrence of diabetes, myocardial infarction, stroke, and cancer: quantifying age patterns in the Dutch population using health survey data
Source: Popul Health Metr. 2011 Sep 1;9:51. doi: 10.1186/1478-7954-9-51 (PMC3175448; doi:10.1186/1478-7954-9-51)
Supplement: Additional file 1 — shows the results if the Bayesian information criterion (BIC) is used to find the optimal smoothing parameters for the P-splines. [file 1478-7954-9-51-S1.PDF]

Appendix A: results using Bayesian Information Criterion

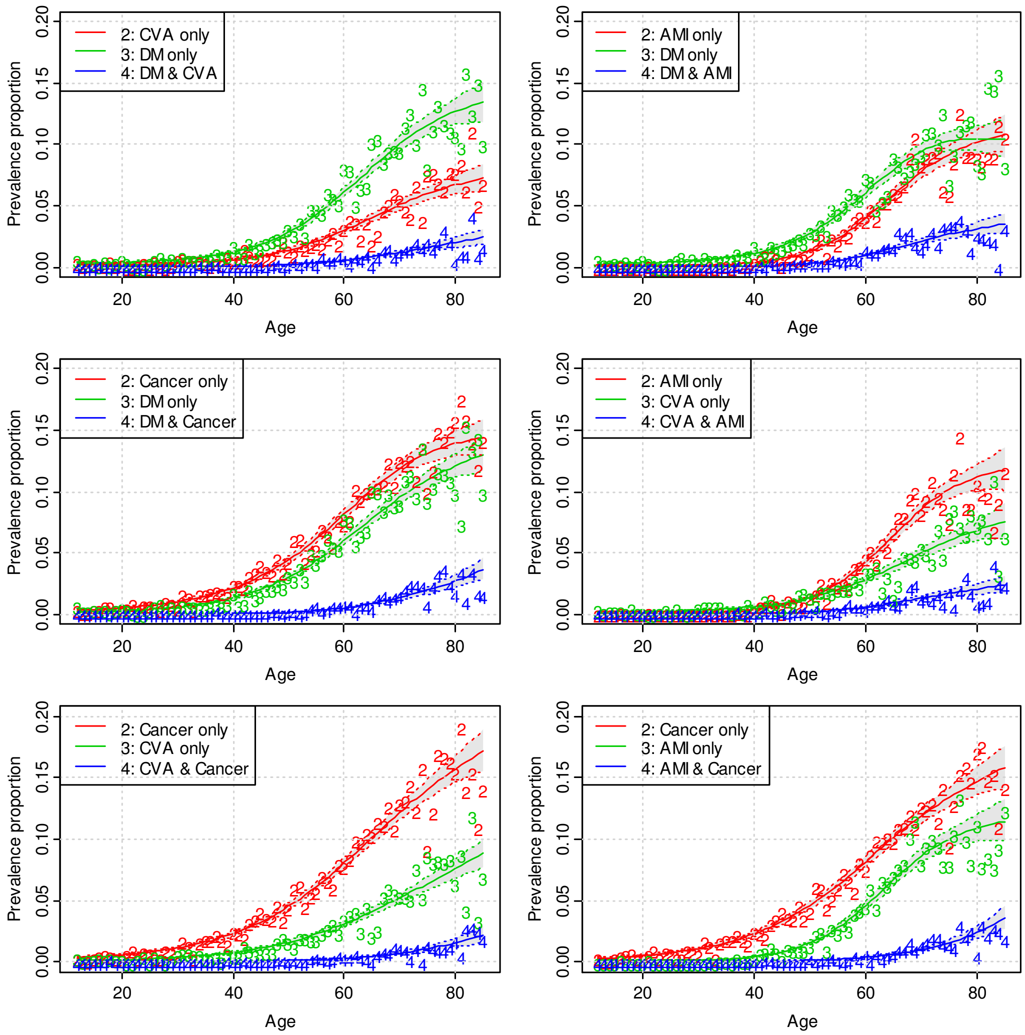

Figure A1: model predictions and data as a function of age (numbers indicate age-specific proportions observed in a particular year in the POLS survey)

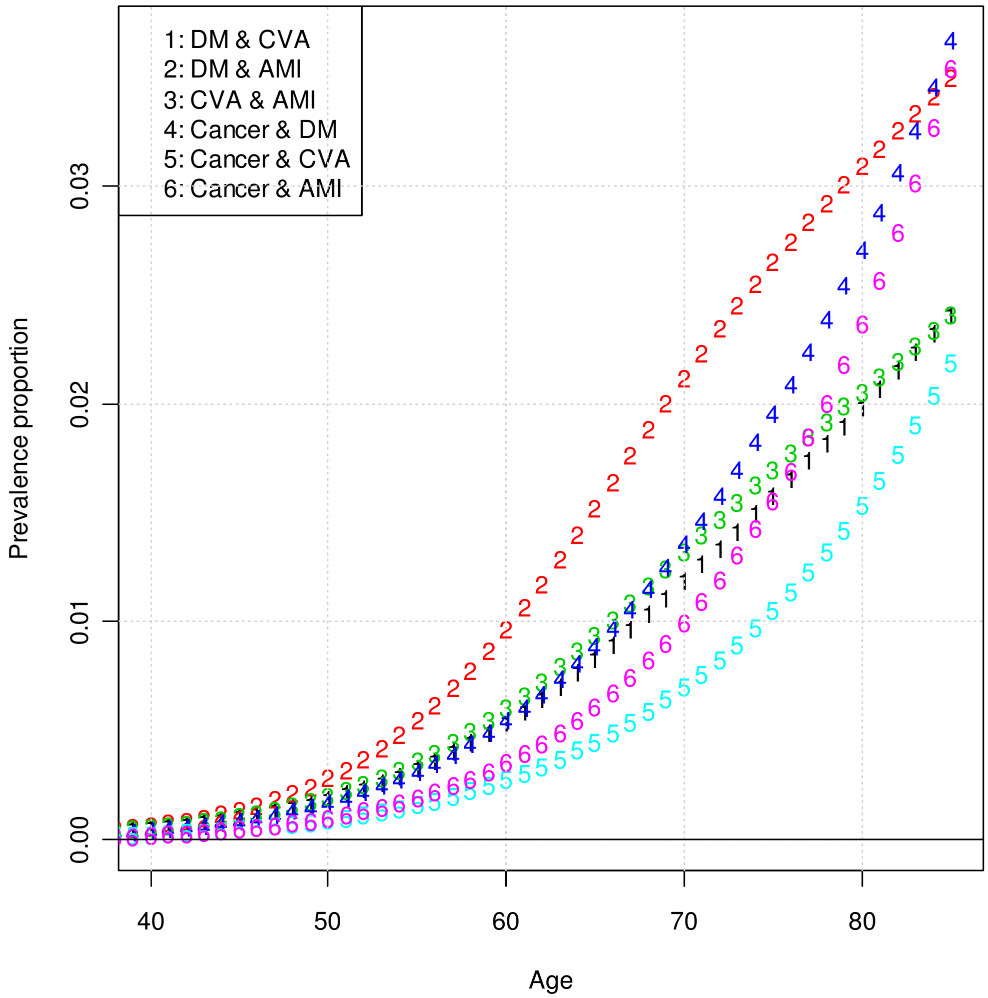

Figure A2: model predictions of prevalence proportion of all pairs of diseases specified by age

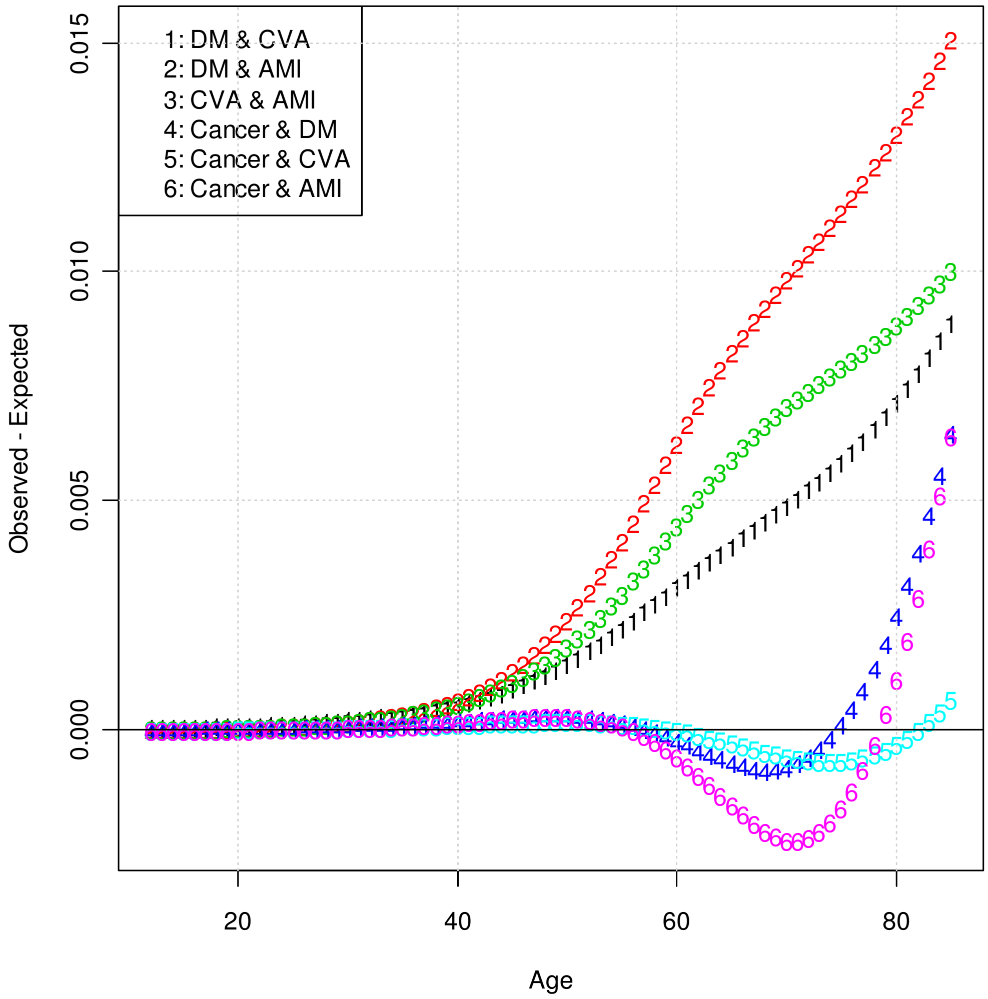

Figure A3: model predictions of observed disease pair prevalence minus expected disease pair prevalence

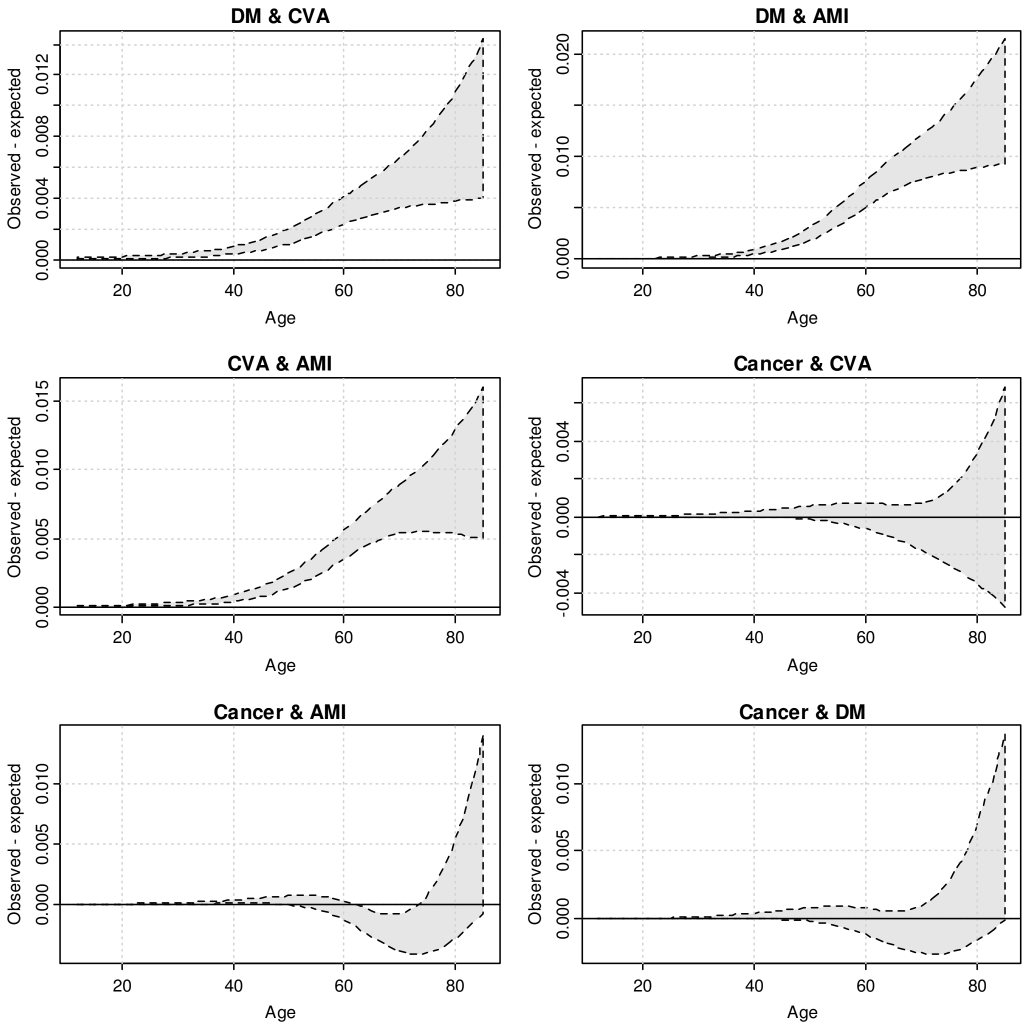

Figure A4: confidence intervals for observed minus expected co-occurrence

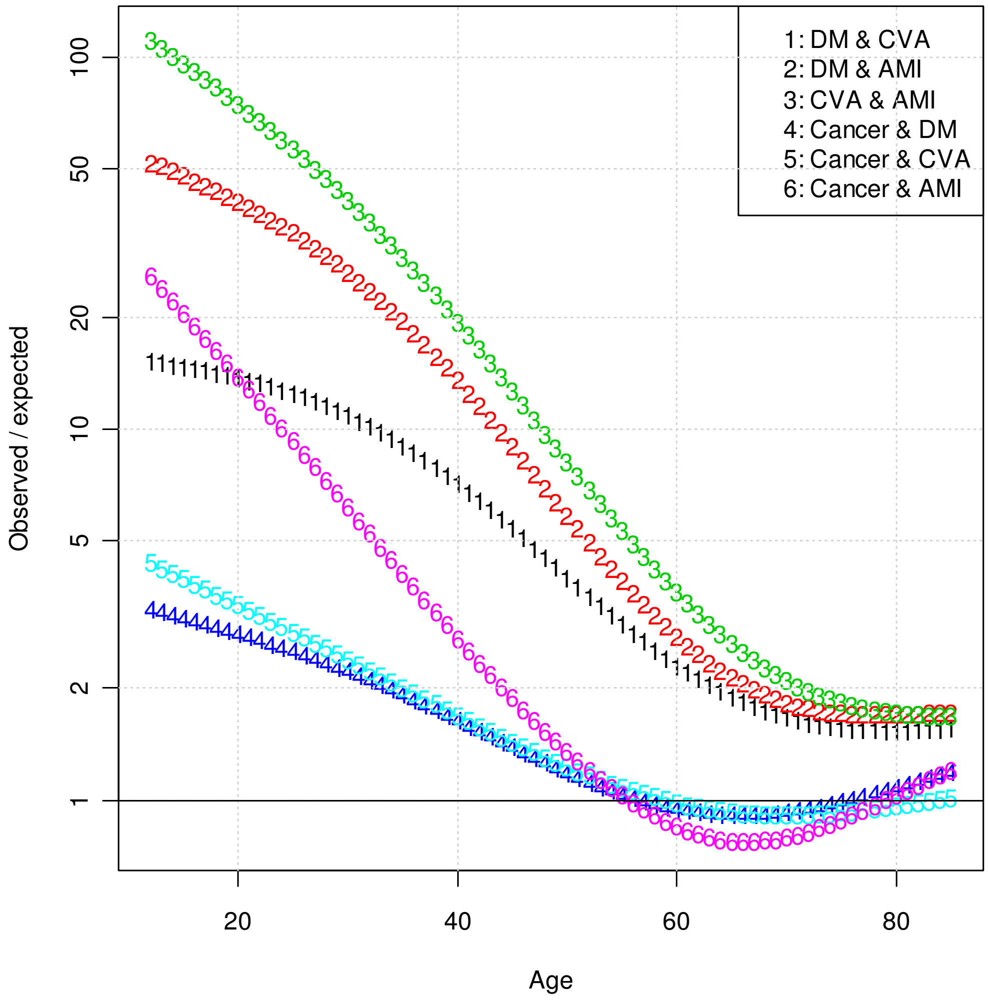

Figure A5: model predictions of observed/expected co-occurrence ratios

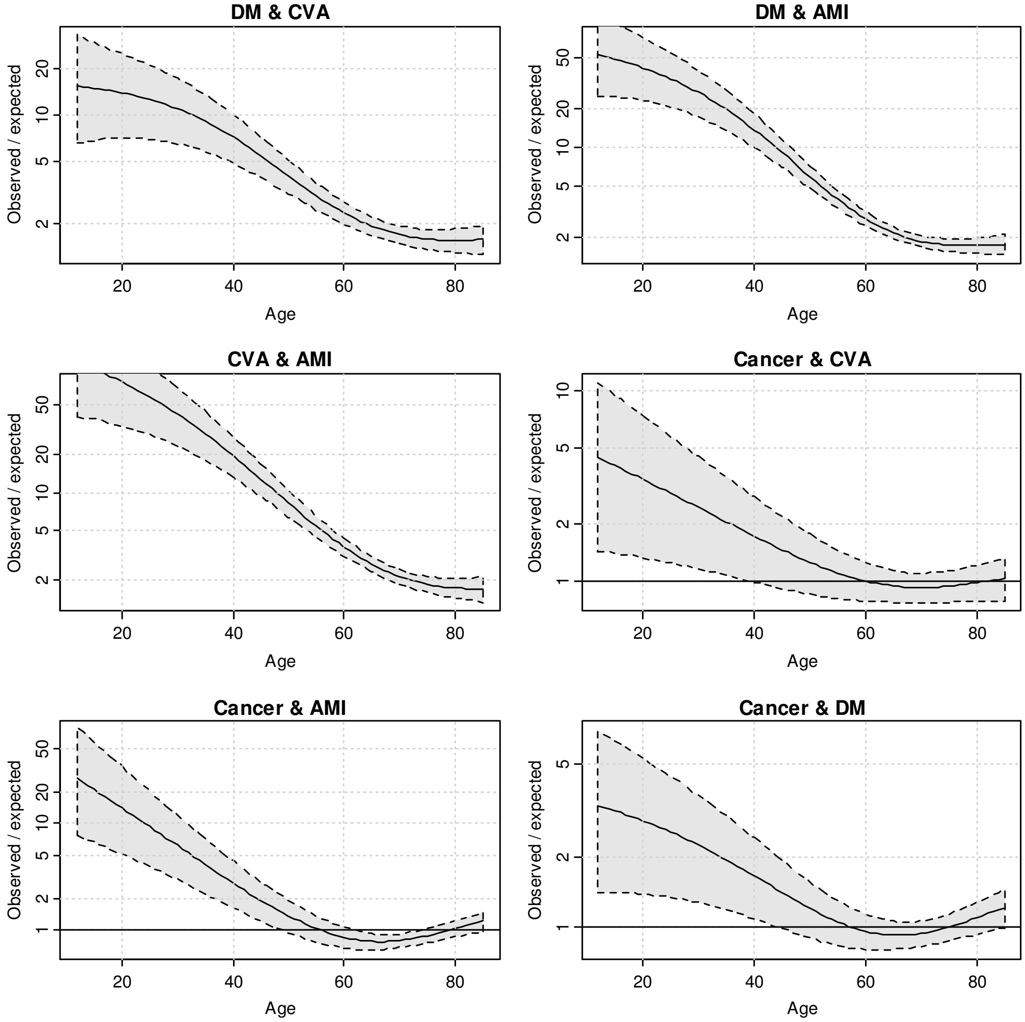

Figure A6: model predictions of confidence intervals observed/expected co-occurrence ratios
